# Identification and Quantification of Flavonoids in Okra (*Abelmoschus esculentus* L. Moench) and Antiproliferative Activity In Vitro of Four Main Components Identified

**DOI:** 10.3390/metabo12060483

**Published:** 2022-05-26

**Authors:** Jing Yang, Xiaoqi Chen, Shuaiqi Rao, Yaochen Li, Yunxiang Zang, Biao Zhu

**Affiliations:** Key Laboratory of Quality and Safety Control for Subtropical Fruit and Vegetable, Ministry of Agriculture and Rural Affairs, Collaborative Innovation Center for Efficient and Green Production of Agriculture in Mountainous Areas of Zhejiang Province, College of Horticulture Science, Zhejiang A & F University, Hangzhou 311300, China; yangjing@zafu.edu.cn (J.Y.); xiaoqichen9528@163.com (X.C.); raoshuaiqi@163.com (S.R.); liyaochen@stu.zafu.edu.cn (Y.L.); yxzang@zafu.edu.cn (Y.Z.)

**Keywords:** *Abelmoschus esculentus*, flavonoids, HPLC-MS/MS, antiproliferative activity

## Abstract

Okra is a kind of flavonoid-rich food which was reported to have a variety of health functions. Flavonoids are the major polyphenolic compounds in okra and are thought to play a role in reducing the risk of disease. The aim of this study was to isolate and identify the flavonoids composition in okra pods and explore the activity of the main flavonoids components identified on inhibiting tumor cell proliferation in vitro. Six individual flavonoids were identified by HPLC-MS/MS: quercetin-3-gentiobioside (Q3G), quercetin-3-sambubioside (Q3S), rutin, quercetin-7-glucoside (Q7G), isoquercitrin (ISO) and quercetin-3-malonylglucoside (Q3M), which were all separated well within 30 min. The analytical method was validated by the recovery of spiked samples and so on. Moreover, four main flavonoids components, namely Q3G, Q3S, ISO and Q3M, exhibited significant (*p* < 0.05) inhibition of NCI-N87, A375, A549 cells proliferation (25−100 μmol/L) and of HFLS-RA (200−300 μmol/L) in different levels, according to MTT method, respectively. It is demonstrated that the flavonoids components of okra exhibited a noteworthy development prospect as a possible nutraceutical dietary supplement.

## 1. Introduction

Long-term eating of flavonoid-rich foods has been associated with reduced risk for certain pathogens and a number of health benefits [1,2], such as resisting bacterial and viral infections, attenuating oxidative stress, cytoprotection, preventing platelet aggregation, cardiovascular and age-related neurodegenerative diseases prevention [3,4], and reducing the Alzheimer’s associated symptoms [5,6].

Okra (*Abelmoschus esculentus* L. Moench) is an annual plant and is believed to originate near Ethiopia in Africa [7,8], which was used for both medicinal and culinary purposes. A variety of health functions of okra, such as reducing blood-sugar and fat [9,10], anti-fatigue [11], antioxidant capacity and reducing inflammatory response in ethanol-induced acute gastricmucosal injury and providing neuroprotection in neurodegeneration diseases such as Alzheimer’s disease have been reported in recent years [6,11,12].

Flavonoids are a kind of polyphenolic compound widely distributed in all vascular plants and also the major polyphenolic compounds in okra [13,14]. The structure of flavonoids is complicated and diversified, but has a similar phenyl benzopyran skeleton. Two aromatic rings are connected by a tetrahydropyran ring to form a series of compounds [15,16]. According to differences of the pyran ring, flavonoids are classified into six major subgroups: flavonols (e.g., quercetin, rutin, kaempferol and quercetagetin), flavanones (e.g., hesperidin, naringin, and naringenin), flavones (e.g., apigenin), flavanols (e.g., epicatechin), isoflavons (e.g., genistein, daidzein) and anthocyanins (e.g., cyanidins) [17,18]. The main flavonoids compounds of okra are quercetin and its derivatives [14].

Flavonoids are thought to have a role in the regulation of canceration at the tumor-promoting phase [19]. According to Yin et al. [20], quercetin (glucoside)-rich foods were predominantly anti-carcinogenic by targeting glycolysis, mainly as β-glycosides. Quercetin can largely inhibit the development of prostate cancer [21], enhance apoptosis of ovarian cancer cells and ameliorate kidney interstitial fibrosis and macrophage accumulation in the kidneys with obstructive nephropathy [22,23]. Reyes-Farias and Carrasco-Pozo [24] showed that the effect of quercetin on glucose metabolism and cellular energy production contributed to its effect on cell viability reduction, metastasis inhibition, and apoptosis induction in cancer cells. Chen et al. [25] found that isoquercitrin inhibited human pancreatic cancer progression in vivo and in vitro, and a therapeutic dose of isoquercitrin significantly inhibited proliferation in pancreatic cancer cells and promoted apoptosis. Moreover, isoquercitrin is a possible candidate as novel cancer chemotherapeutics that targets caspases, which play essential roles in programmed cell death by cleaving cellular proteins at specific aspartate residue [25], and was found to reduce the production of interleukin-6 in human osteosarcoma MG-63 cells stimulated by tumour necrosis factor α [26]. Flavonoids from okra flower exerts significant antitumor efficacy on the inhibition of colorectal cancer cell proliferation and metastasis as well as tumour growth in vivo [27]. The anticancerous effect of okra seed extracts have also been documented in vitro through human-derived breast cancer cells (MCF-7), and the flavonoid isoquercitrin, in asynergistic association with other flavonoids, results in the apoptosis of cancerous cells [28]. Quercetin-3-O-β-D-galactoside, which is an important flavonoid constituent of okra, has also been reported for its anticancerous potential in gastric cancer cells (CHI) [29,30].

Previous studies have also suggested that daily and constant ingestion of flavonoids through okra might reduce the risk of developing certain types of cancer such as colon cancer, prostate cancer, breast cancer and liver cancer [21,31]. Zhao et al. [32] found that flavonoid-rich extracts from okra flowers exert antitumor activity in colorectal cancer. However, the scientific reports on their anticancer activity are still sparse. The quantitative determination and antitumor properties investigation of flavonoids in okra should be of great importance to both scientific research and commercial application.

The aim of this study was to isolate and identify flavonoids in okra pods and tentatively explore the effects of the main flavonoids of okra on inhibiting several tumor cell lines proliferation in vitro, which could promote the development and application of this for further medicinal and edible purposes.

## 2. Results

### 2.1. Identification of Flavonoids in Okra Pod

#### 2.1.1. Six Individual Flavonoids Were Identified

**Figure 1:**
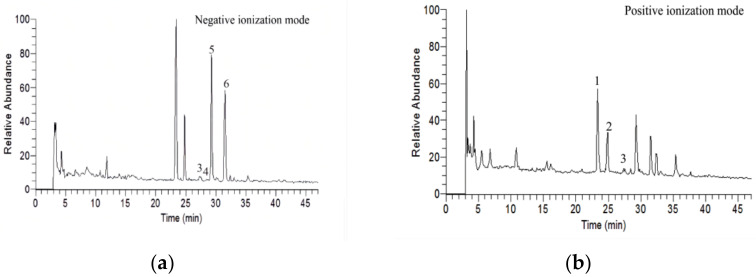
TIC chromatogram of flavonoids composition from the extracts of okra. (**a**) Negative ionization mode. (**b**) Positive ionization mode.

**Figure 2:**
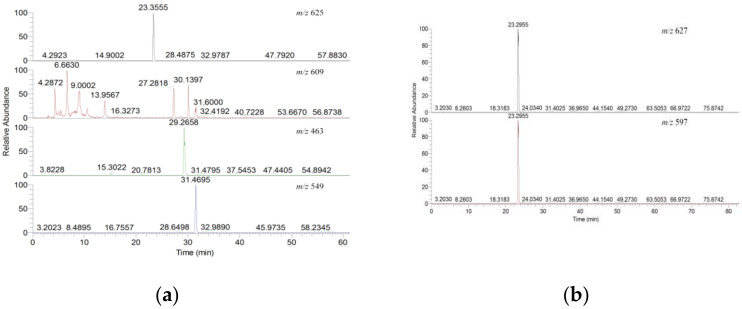
Screening of flavonoids through HPLC-MS/MS from extracts of okra. (**a**) negative ion scan (*m*/*z* 625; *m*/*z* 609; *m*/*z* 463; *m*/*z* 549). (**b**) positive ion scan (*m*/*z* 627; *m*/*z* 597).

The flavonoids composition from the hydro-ethanol extract of okra were analyzed by HPLC-MS in negative and positive ionization modes (Figure 1), and followed by a high resolution MS for molecular formula determination, through negative ion scanning (*m*/*z* 625; *m*/*z* 609; *m*/*z* 463; *m*/*z* 549) and positive ion scanning (*m*/*z* 627; *m*/*z* 597), to identify the listing of precursor ions and distinguish isomers based on a listing of potential targets (Figure 2). Seven candidate components were provisionally identified via their *m*/*z* values, and literature data, namely quercetin-3-gentiobioside (Q3G), quercetin-3-diglucoside (Q3D), quercetin-3-sambubioside (Q3S), rutin, quercetin-7-glucoside (Q7G), isoquercetin (ISO) and quercetin-3-malonylglucoside (Q3M), which respectively correspond to peak 1 (Q3G, Q3D or mixture), peak 2 (Q3S), peak 3 (rutin), peak 4 (Q7G), peak 5 (ISO) and peak 6 (Q3M). Among that, peak 1 could be Q3G, Q3D or mixture, and all the other five peaks are have only one candidate.

All of the seven candidate flavonoids standard substances were purchased, and identification of peaks was then confirmed by spiking samples with standard one by one as further confirmation of the veracity of the identifications. Flavonoids standard solution exhibited clearly characteristic UV/Vis spectrum, with maximum absorbance in the range of 250–260 nm and 340–370 nm (Figure S1). Based on the characteristic spectrums and our previous studies, 256 nm was chosen to determine the flavonoids profiles in this experiment.

Fortunately, Peak 1 was identified as Q3G, and the possibility of mixture was excluded due to Q3D standard being washed out earlier during chromatographic separation. The candidate components of Peak 2 to 6 were confirmed after comparing their retention times and spectra to those of relative flavonoids standards. Finally, six individual flavonoids were identified (Table 1).

**Figure 3:**
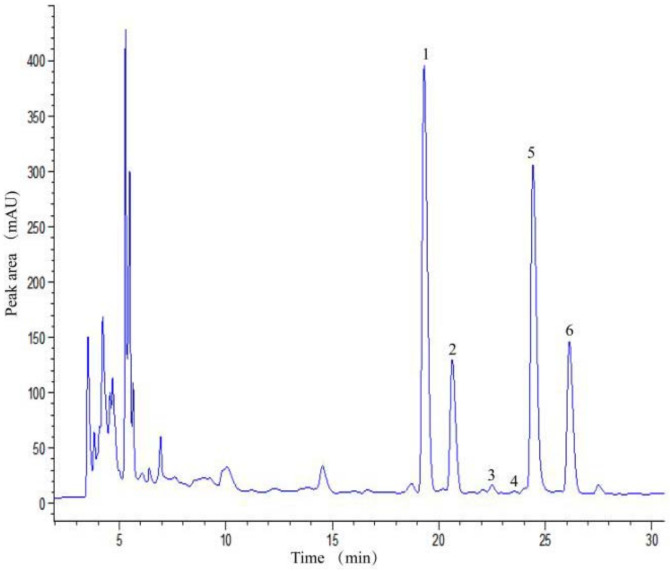
HPLC chromatogram of okra flavonoids (1. Q3G; 2. Q3S; 3. Rutin; 4. Q7G; 5. ISO; 6. Q3M).

In this study, Q3G (52%) was the most abundant individual flavonoid, followed by ISO (27%), Q3S (11%), Q3M (7%), rutin (2%), Q7G (1%) in that order (Figure 3) and the total flavonoids content in the pod of okra was range of 4126.64−4873.55 μg/g DW.

#### 2.1.2. Method Verification

To ensure the availability and accuracy of the current method, a series of relative parameters were checked and calculated (Table 2). High intra-day and inter-day precisions were achieved with relative standard deviation (RSD) values, which were 0.72−2.26% and 0.95−3.72%, respectively. The recovery of spiked samples was found between 96.53% and 102.54%, and the correlation coefficient was above 0.9995. Otherwise, the limit of detection (LOD) values was between 0.045 and 0.760 μg/mL, while the limit of quantification (LOQ) values was between 0.152 and 2.535 μg/mL. The upper limit linear ranges of the tested compounds were measured among 100 to 1000 μg/mL according to the concentration range of different individual flavonoids in samples. The current analytical method can be successfully applied to qualitative and quantitative analysis of six flavonoids in okra.

### 2.2. Antiproliferative Activity In Vitro of the Four Main Individual Flavonoids in Okra Pod

**Figure 4:**
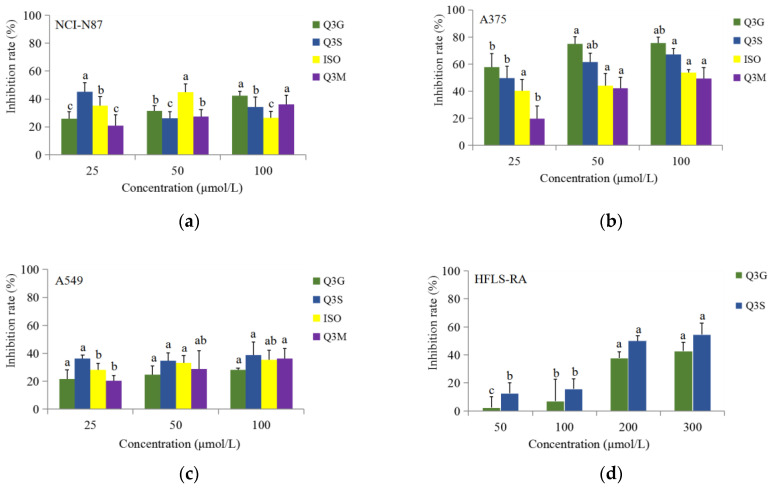
Anti-proliferative activity of flavonoids in NCI-N87 (**a**), A375 (**b**), A549 (**c**) and HFLS-RA cell line (**d**) by MTT assay (Q3G: quercetin-3-gentiobioside, Q3S: quercetin-3-sambubioside, ISO: isoquercetin, Q3M: quercetin-3-malonylglucoside; Different letters indicate significant difference between concentrations (*p* < 0.05)).

The antiproliferative activity of the main okra flavonoids components was tentatively explored in vitro for the inhibition of cell growth in NCI-N87, A375, A549 and HFLS-RA cell lines (Figure 4). Q3G, Q3S, ISO and Q3M were selected for in vitro antiproliferative activity assay due to the fact that they were the major flavonoids in okra (accounting for more than 96% of the total flavonoids). The test concentration ranges of four flavonoids components were 25−100 μmol/L (NCI-N87, A375and A549) and 0−300 μmol/L (HFLS-RA), respectively. The concentration range was selected according to the literature review and the concentration of flavonoids in okra was determined.

The results show that the NCI-N87 and A375 cells were the most sensitive tumor cell lines. The maximum inhibition of NCI-N87 cells proliferation of four major okra flavonoids were demonstrated as 42.45% (100 μmol/L Q3G), 45.20% (25 μmol/L Q3S), 44.73% (50 μmol/L ISO) and 36.13% (100 μmol/L Q3M), respectively (*p* < 0.05) (Figure 4a). Meanwhile, four major okra flavonoids demonstrated an almost maximum inhibition of A375 cells proliferation of 75.99% (Q3G), 67.19% (Q3S), 53.73% (ISO) and 49.35% (Q3M) (*p* < 0.05), respectively, at 100 μmol/L (Figure 4b). In comparison, the highest cell growth inhibitory effect was observed with the substances Q3G and Q3S in A375 cells, and both of their inhibition rates were greater than 50% (25−100 μmol/L) (Figure 4b). Four major okra flavonoids have some anti-proliferation effect on A549, but the difference of anti-proliferation ability at different concentrations was not obvious (Figure 4c). Furthermore, Q3G and Q3S demonstrated a maximum inhibition of HFLS-RA cells proliferation of 42.99% and 54.61% at 300 μmol/L, respectively (*p*< 0.05). The other two flavonoids were not tested at higher concentrations in HFLS-RA cells because of the solubility in water (Figure 4d). In general, all of the four major okra flavonoids (25−100 μmol/L) exhibited significant (*p*< 0.05) inhibition of NCI-N87, A375, A549 cells proliferation (Figure 4a–c), and the Q3G and Q3S (200−300 μmol/L) showed significant (*p*< 0.05) inhibition of HFLS-RA cell proliferation (Figure 4d), and the four tested okra flavonoids had no effects on normal cells (Table S1).

## 3. Discussion

In this work, six individual flavonoids, including Q3G, Q3S, Rutin, Q7G, ISO and Q3M were detected. The results are in agreement with D’Urso et al. [35] and one more flavonoid (Q7G) was identified in our research. In addition, the separation and identification of flavonoids in okra in this study is much further based on previous studies [14,36,37]. Among the six components, Q3G, ISO, Q3S and Q3M were the main individual flavonoids, accounting for more than 96% of the total flavonoid. Q3G was also detected as the most abundant flavonoid of the tested okra fruits in some previous studies [14]. Wu et al. [36] also showed that Q3G and ISO were the main flavonoids in okra, which was in accordance with our results.

The results of MTT experimental observations indicated that four main flavonoids (Q3G, Q3S, ISO and Q3M) had good inhibitory effects on the proliferation of several tumor cell lines in vitro. The inhibition by the four main flavonoids in okra might be associated with their glycoside derivatives [38]. There is a paucity of reports regarding the anticancer effects of the four flavonoids. Cao et al. [39] suggested that quercetin suppressed A375 tumor growth and STAT3 activities in xenografted mice model. The four main flavonoids identified in the current study, quercetin-3-gentiobioside (Q3G), quercetin-3-sambubioside (Q3S), isoquercetin (ISO) and quercetin-3-malonylglucoside (Q3M), all belong to quercetin glycoside derivatives and also show obviously antiproliferative activity in A375 cell line. Quercetin is almost insoluble in water, and mostly exists in plants in the form of glycosides as well as their derivatives [40,41]. Numerous studies have found that substances in the flavonoid group may induce apoptosis of cancer cells [42]. Additionally, some of the case-control studies have indicated an inverse association between intake of flavonoids and cancer risk (lung cancer, upper digestive tract cancer, and gastric cancer) [43,44]. In addition, the different flavonoids have differential effects on cancer cell inhibition, which seems to relate to their different chemical structure, such as the position, number and substitution of the hydroxyl group of the B ring and saturation of the C2-C3 bond [45].

Comparing the effects on cancer cell inhibition of flavonoids detected in this study to the effects on some medicinal plants and other natural sources for drug leads according to literatures, it is demonstrated that the flavonoids in okra could be developed as a possible nutraceutical dietary supplement for anti-proliferation effect. For example, Q3S could be a substance that has some effect on the treatment of gastric carcinoma. The effect of Q3S at post-treatment by 25 μmol/L on the proliferation of NCI-N87 cells was similar to 1 μg/mL trastuzumab + 1 μmol/L apatinib (treat advanced gastric cancer or adenocarcinoma of the gastroesophageal junction) [46]. Furthermore, Q3G (25 μmol/L) and Q3S (50 μmol/L) inhibited the proliferation of A375 cells were similar to that with treatment of 0.4 μmol/L homoharringtonine (used in the treatment of acute myelogenous leukemia) [47]. On the other hand, the four flavonoids inhibited the proliferation of A375 cells was weaker than that treatment with curcumin [48].

## 4. Materials and Methods

### 4.1. Plant Material

The okra (*Abelmoschus esculentus* L. Moench) pods were purchased from local market in Lin’an, Zhejiang Province, which were sorted out for uniformity in shape, size and without any mechanical injuries. The samples were precooled with liquid nitrogen, then lyophilized, and finally ground to make a fine powder. The powders were stored in sealed plastic bags at −20 °C for further analysis.

### 4.2. Identification and Determination of Flavonoids

#### 4.2.1. Sample Solution

The freeze-dried powders of okra pod were weighed precisely and sonicated with 70% ethanol (solid-to-solvent ratio is 1:20g/mL) by ultrasonical extraction at 45 °C for 20 min. The extraction was filtered, transfered to a rotary evaporator bottle and concentrated to drying in a rotary evaporator (Büchi R-215, Flawil, Switzerland) at 45 °C. The condensate was redissolved with 2 mL 70% ethanol to gain the sample solutions after filtered (0.22 μm) for further analysis.

#### 4.2.2. Identification of Flavonoids in Okra

The sample solution was identified by HPLC-MS/MS. Chromatographic analysis was performed in an Agilent 1200 Series HPLC system (Agilent Technologies, Wilmington, DE, USA) equipped with a diode array detector, using a SpherisorbInertsil ODS-SP (5 μm, 4.6 mm × 250 mm) (GL Sciences, Tokyo, Japan). The column temperature was maintained at 30 °C by a thermostat with a flow rate of 0.8 mL/min. The mobile phase was composed of A (1% acetic acid glacial, *v*/*v*) and B (acetonitrile) with a gradient elution: 0–1 min 10%B; 1–40 min 10–30%B. The injection volume was 10 μL.

Mass spectrometric detection was carried out using a Finnigan LCQ Deca XP Max (Thermo, San Jose, CA, USA) equipped with an electrospray ionization (ESI) source. The parameters in the source were set as follows: capillary voltage of 26 V, probe temperature of 375 °C, N_2_ as drying gas and MS Scan from 100 to 1800 *m*/*z*. Mass spectra were obtained in both positive and negative ionization modes. Data acquisition was carried out with the Xcalibur^®^ data system (Thermo, San Jose, CA, USA).

To further confirm the identified components, the standard substances of all of the seven candidate flavonoids were purchased, that is quercetin-3-gentiobioside (Q3G), quercetin-3-diglucoside (Q3D), quercetin-3-sambubioside (Q3S), rutin, quercetin-7-glucoside (Q7G), isoquercetin (ISO) and quercetin-3-malonylglucoside (Q3M), from Shanghai Yuanye Bio-Technology Co., Ltd. (Shanghai, China) and Sigma-Aldrich (St. Louis, MO, USA), respectively. Subsequently, the components were finally identified according to the retention time of the comparison standard and recovery test.

#### 4.2.3. Quantitative Analysis of Flavonoids in Okra

The flavonoids in okra were quantified by external standard method. Quantification of the mixed reference solution was performed by Agilent 1200 Series HPLC/DAD using five point regression curves (R^2^ > 0.999) (Table 1). Chromatographic analysis refers to HPLC-MS/MS analysis. The working standard mixture was gained by dissolving all of the standard substances in 70% ethanol and diluting with ethanol to a series of appropriate concentrations and used to construct calibration curves. The concentration ranges for six analytes were unfolded in Table 2. All the solutions were injected after being filtered by a 0.22 µm filter.

The accuracy of the above method was verified by a recovery test, which was performed using the above method by six (*n* = 6) replicate injections of spiked samples with a ratio of 1:1 between the standard content and the sample content and extracted. Precision/injection repeatability test was performed by six (*n* = 6) replicates injections of the mixed reference solution. The intra-day variations were injected in 1 day; the inter-day variations were injected after a 1 day interval. Variations were expressed as the percentage relative standard deviation (RSD%) of the replicates.

### 4.3. Determination of Antiproliferative Activity

#### 4.3.1. Cell Culture

The tumor cell lines, including melanoma cell lines (A375), type II epithelial lung carcinoma cells (A549), gastric cancer cell lines (NCI-N87), and the normal human cells fibroblast-like synoviocyte rheumatoid arthritis (HFLS-RA) were obtained from Shanghai Cells Bank, Institutes of Life Sciences of the Chinese Academy of Sciences.

The cells were treated according to the manufacturer’s instructions provided by Shanghai Cells Bank, Institutes of Life Sciences of the Chinese Academy of Sciences. A total of 10% fetal bovine serum (FBS, Sigma-Aldrich, Steinheim am Albuch, Germany) and antibiotics (100 U/mL penicillin and 100 µg/mL streptomycin) were added as indicated. The cells were maintained in sterile carbon dioxide incubator in a humidified atmosphere of 95% air, 5% CO_2_ at 37 °C.

#### 4.3.2. Antiproliferative Activity

Pure substances of the four major flavonoids, namely Q3G, Q3S, ISO and Q3M, were selected for in vitro antiproliferative activity assay. Four concentration levels of Q3G and Q3S were set (50, 100, 200, 300 μmol/L) to HFLS-RA cell line, while three levels of Q3G, Q3S, ISO and Q3M were set (25, 50, 100 μmol/L) to NCI-N87, A375, and A549 cell lines. The normal human colon cell CCD-18CO was used as control. The antiproliferative activity of them was analyzed according to the MTT method previously described with slight modifications [49]. Briefly, four cell lines were plated at a concentration of 5000 cells per well in 96-well plates and cultured for 24 h, respectively. All of the cell lines were treated with 20 μL samples at the indicated doses for 48 h (Repeat 7 times for each set). Then, the medium was incubated at 37 °C for 4 h with 180 μL 5% FBS-containing medium and MTT reagent (20 μL, 0.5 mg/mL final concentration in medium). After the media were removed, 150 μL DMSO (dimethyl sulfoxide) was added to each well. Subsequently, optical density (OD) values at 492 nm were recorded in a microplate reader. The control group was set without any of the four flavonoids. The inhibition rate of cell proliferation determined by MTT assay was expressed as:

Growth inhibition rate (%) = (1-Absorbance of experimental group/Absorbance of blank control group) × 100%.

### 4.4. Statistical Analysis

All results were expressed as means ± standard deviation (SD). Statistically significant differences among groups were determined using one-way analysis of variance (ANOVA) (SPSS software version 25.0), *p* < 0.05 confidence level was considered significantly different.

## 5. Conclusions

The present study exhibited a reliable and effective method for flavonoids determination in okra and might also be utilized for the determination of flavonoids contained in other plants as a reference. Okra is rich in flavonoids and Q3G, Q3S, ISO and Q3M had good antiproliferative activity in vitro. At present, it is still lack of clinical evidence of cancer prevention and treatment in human from okra. This experiment provides some preliminary results and the mechanisms of action are still unclear and need deep research.

## Figures and Tables

**Table 1:** Flavonoids compounds detected and characterized based on HPLC with HRMS analysis in positive and negative ionization modes.

Peak	Tentative Assignment	tR (min.)	[M+H]^+^ (*m*/*z*)	[M H]^−^ (*m*/*z*)	Molecular Formula		MS/MS Fragment Ions	References
1	Quercetin-3-gentiobioside	18.185	626.7747	-	C_27_H_30_O_17_	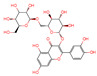	303.1241	-
2	Quercetin-3-sambubioside	19.874	596.7314	-	C_26_H_28_O_16_	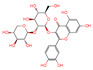	303.1179	[14]
3	Rutin	21.484	610.7375	609.1411	C_27_H_30_O_16_	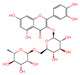	301.1387	[14]
4	Quercetin-7-glucoside	22.248	-	463.0782	C_21_H_20_O_12_	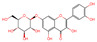	-	-
5	Isoquercitrin	23.815	-	463.0837	C_21_H_20_O_12_	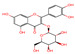	301.1503	[33,34]
6	Quercetin-3-malonylglucoside	25.194	-	548.6969	C_24_H_22_O_15_	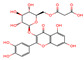	504.9665	[14]

**Table 2:** Linearity, Precision and Accuracy Results for Tested Compounds.

No.	Flavonoids Standards	Calibration Equations	Correlation Coefficient (R^2^)	LOD (μg/mL)	LOQ (μg/mL)	Linear Range (μg/mL)	RSD% ^1^ (Intra-Day)	RSD% ^2^ (Inter-Day)	The Recovery of Spiked Sample REC% ^3^
1	Quercetin-3-gentiobioside	y = 14.66x −1 0.596 ^4^	1	0.204	0.680	0–800	0.72	0.95	96.83
2	Quercetin-3-sambubioside	y = 20.461x − 24.366	0.9999	0.120	0.400	0–400	1.88	2.33	102.54
3	Rutin	y = 10.342x + 4.8446	0.9999	0.209	0.696	0–100	1.75	3.46	96.60
4	Quercetin-7-glucoside	y = 5.8708x − 1.3649	1	0.106	0.355	0–100	2.04	3.72	98.93
5	Isoquercitrin	y = 34.06x + 57.405	0.9995	0.045	0.152	0–1000	1.60	2.93	96.53
6	Quercetin-3-malonylglucoside	y = 27.904x + 149.3	0.9997	0.760	2.535	0–1000	2.26	2.38	101.21

^1^ Values are means of intra-day assays (*n* = 6). ^2^ Values are means of inter-day assays (*n* = 6).^3^ Values are means of the recovery of spiked sample (*n* = 6). ^4^ Chromatographic peak area (denoted by the symbol) versus concentration (denoted as x) where x was in μg/mL.

## Data Availability

The data presented in this study are available on request from the corresponding author. The data are not publicly available due to privacy.

## Supplementary Materials

The following supporting information can be downloaded at: https://www.mdpi.com/article/10.3390/metabo12060483/s1, Figure S1: Typical UV/Vis spectra of the different flavonoids standards identified. Table S1: Inhibitory effects of the flavonoids components on normal cells.
