# Supplementary material for: Identification and Quantification of Flavonoids in Okra (Abelmoschus esculentus L. Moench) and Antiproliferative Activity In Vitro of Four Main Components Identified
Source: Metabolites. 2022 May 26;12(6):483. doi: 10.3390/metabo12060483 (PMC9228595; doi:10.3390/metabo12060483)
Supplement: Supplementary file 1 [file metabolites-12-00483-s001.zip › Supplementary Material_Table S1.pdf]

Table S1: Inhibitory effects of the flavonoids components on normal cell CCD-18CO (normal human colon cells).

Flavonoids components	Inhibition growth efficiency (%) Concentration(μmol/L)			
	25	50	100	200
Q3G	2.52±0.22	1.35±0.31	3.71±1.20	1.51±0.32
Q3S	-3.62±1.35	2.39±0.53	2.49±0.87	2.72±1.02
ISO	4.84±1.34	-1.47±0.52	4.52±1.40	3.02±1.43
Q3M	1.25±0.46	3.52±0.38	-2.27±0.82	2.13±0.72

Data represent the means ± SD from three independent experiments.
