# Supplementary figures and images for: Identification and Quantification of Flavonoids in Okra (Abelmoschus esculentus L. Moench) and Antiproliferative Activity In Vitro of Four Main Components Identified
Source: Metabolites. 2022 May 26;12(6):483. doi: 10.3390/metabo12060483 (PMC9228595; doi:10.3390/metabo12060483)

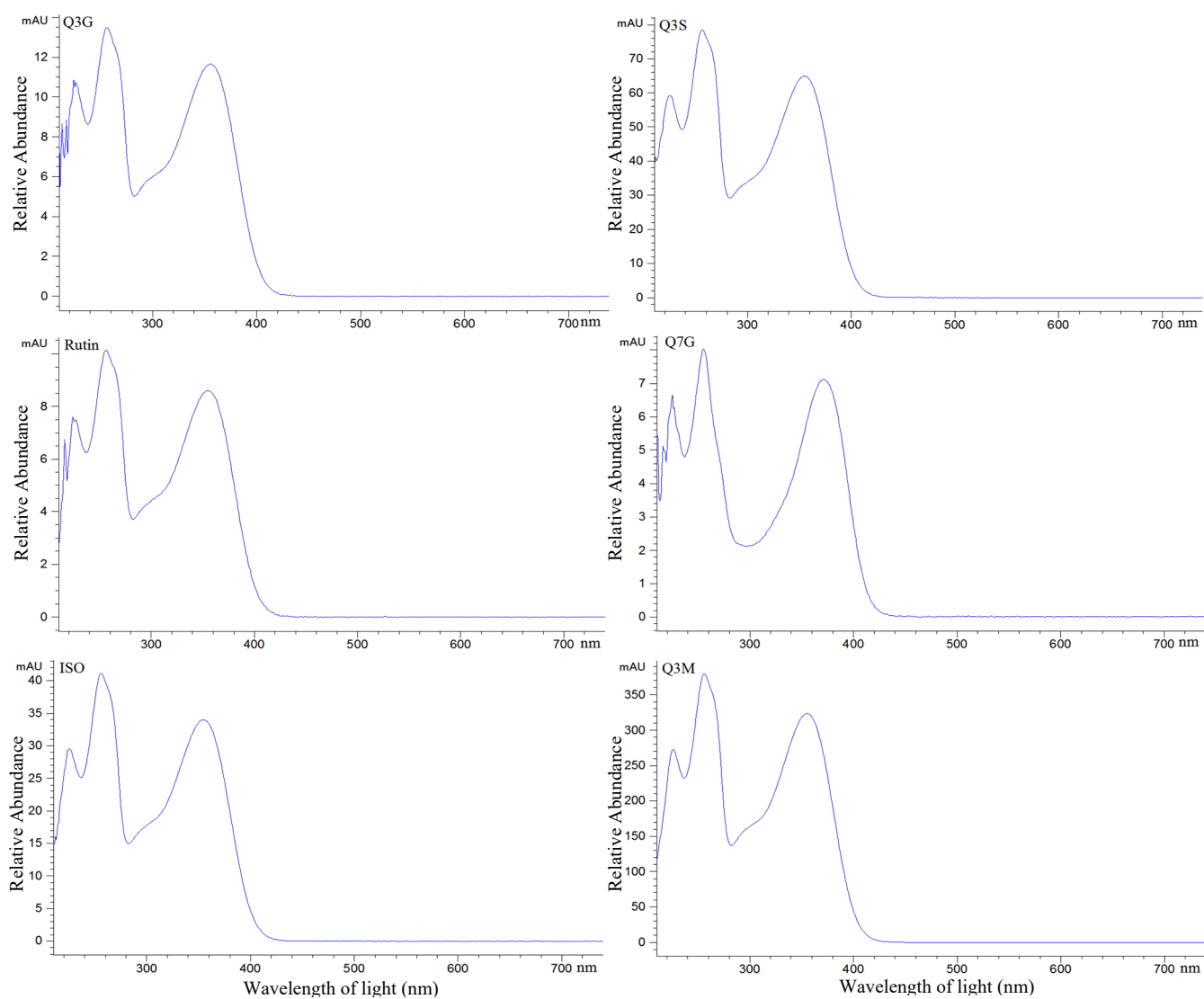

**Figure S1.** Typical UV/Vis spectra of the different flavonoids standards identified.

Supplement: Supplementary file 1 [file metabolites-12-00483-s001.zip › Supplementary Material_Figure S1.pdf]
